# Selection of New Probiotics for Endometrial Health

**DOI:** 10.3389/fcimb.2019.00114

**Published:** 2019-04-17

**Authors:** Empar Chenoll, Inmaculada Moreno, María Sánchez, Iolanda Garcia-Grau, Ángela Silva, Marta González-Monfort, Salvador Genovés, Felipe Vilella, Cristina Seco-Durban, Carlos Simón, Daniel Ramón

**Affiliations:** ^1^Biopolis SL/Archer Daniels Midland, R&D Department, Valencia, Spain; ^2^Igenomix Foundation, Instituto de Investigación Sanitaria Hospital Clínico (INCLIVA), Valencia, Spain; ^3^Igenomix SL, Research Department, Paterna, Spain; ^4^Department of Obstetrics and Gynecology, University of Valencia, Valencia, Spain; ^5^Ferring Pharmaceuticals, Madrid, Spain; ^6^Department of Obstetrics and Gynecology, School of Medicine, Stanford University, Palo Alto, CA, United States

**Keywords:** *L. rhamnosus* BPL005 (CECT 8800), endometrial health, gynecological pathogens, probiotic, pathogen inhibition

## Abstract

Microbiota is a crucial player in gynecologic health, in which bacteria can shift to a dysbiotic state triggering a pathogenic process. Based on an ecological understanding of the problem, the aim of this study is to select a potential probiotic strain to improve female reproductive tract based on its capacity to initially lower pH and to promote the reduction of pathogenic bacteria. Based on this rationale, strain *Lactobacillus rhamnosus* BPL005 was initially selected for its capacity to reduce *in vitro* pH levels and produce organic acids. Subsequently, strain *L. rhamnosus* BPL005 (CECT 8800) was demonstrated to have a protective role on endometrial infections in an *in vitro* model of bacterial colonization of primary endometrial epithelial cells with *Atopobium vaginae, Gardnerella vaginalis, Propionibacterium acnes*, and *Streptococcus agalactiae*. In this model, BPL005 when co-cultured with those pathogens was shown to lower pH and to produce organic acids, being lactic acid the most relevant. The co-cultivation of strain *L. rhamnosus* BPL005 with tested reference pathogens produced a significant reduction in *P. acnes* and *St. agalactiae* levels and a non-significant reduction in *A. vaginae* and *G. vaginalis*. The colonization of *L. rhamnosus* BPL005 in the culture decreased IL-6, IL-8, and MCP-1, heightened in the presence of pathogens, and increased IL-1RA and IL-1 beta. Finally, safety was evaluated showing no signs of cytotoxicity, irritation in vaginal tests, or allergic contact dermatitis potential through the Local Lymph Node Assay. Overall, these results show the potential of *L. rhamnosus* BPL005 strain as a probiotic in gynecological health.

## Introduction

There is increasing evidence for microbiota as a key player not only in gynecologic health but also in pregnancy and infant cycles. The female reproductive tract microbiome has mainly been described based on studies analyzing vaginal samples. Although there is a variation among different ethnicities (Gajer et al., 2012) and also an evolution throughout the menstrual cycle (Ravel et al., 2011), it is well-established that lactobacilli dominate the healthy vagina (Hill, 1993). These studies have shown that healthy and asymptomatic women present vaginal microbiomes dominated by different species of *Lactobacillus* belonging to community sate types (CST) I, II, III, and V (*L. crispatus, L. iners, L. jensenii*, and *L. gasserii*, respectively) while women suffering from bacterial vaginosis (BV) present a significant increase in other genera of bacteria as *Atopobium, Prevotella*, or *Megasphaera* to the detriment of lactobacilli included in CST-IV (Ravel et al., 2011). In this study, vaginal microbiome was divided in five biotypes, being four of them dominated by lactobacilli and considered as healthy (pH < 5.0) and defining the group IV with higher pH (pH > 5.0) and higher diversity. In a healthy status, microbiota is balanced and forms a stable ecological unit dominated by *Lactobacillus* species, which fixes pH below 5.0 and controls non-desirable groups. In a pathogenic process, the microbiome shifts to a dysbiotic state, in which lactobacilli drop, pH is increased to values above 4.5 and other groups such as *Gardnerella, Atopobium, Prevotella*, and *Streptococcus*, among others, can overgrow (Srinivasan et al., 2012). Short chain fatty acids (SCFAs) are a product of anaerobe growth, which include acetate and propionate, together with a depletion of lactic acid due to lower lactobacilli cell counts (Yeoman et al., 2013). Along with their ability to lower the pH, *Lactobacillus* strains have other potential characteristics that can exert control over unbalanced microorganisms such as the production of hydrogen peroxide (Sgibnev and Kremleva, 2015), bacteriocins (Ocaña et al., 1999b; Aroutcheva et al., 2001), and their capacity of adhesion (Coudeyras et al., 2008).

Several studies have demonstrated there is bacterial colonization beyond the vagina, showing that the upper reproductive tract is not sterile (Baker et al., 2018). Recently, a study surveying the female reproductive tract confirmed the existence of a microbiota continuum starting in the vagina and progressing to the deepest organs in the tract—cervix, uterus, tubes, ovaries, and even colonizing the pouch of Douglas—in women with non-infectious conditions (Chen et al., 2017).

Altogether, these results emphasize the role of microbiota in reproductive health and also the concept of the reproductive tract as a relatively stable ecological system with the presence of *Lactobacillus*.

In a recently published work, our research group described that the endometrial microbiota of infertile patients subjected to assisted reproductive technology can be classified as LD (≥90% *Lactobacillus* spp.) and NLD (<90% *Lactobacillus* spp.) (Moreno et al., 2016). This classification helps predict reproductive success in terms of implantation, pregnancy and live birth rates, showing that an NLD microbiota (specially with presence of *Gardnerella* or *Streptococcus*) is strongly associated with adverse reproductive outcomes when compared to subjects presenting an LD endometrial microbiota. This is consistent with the observation that patients with live births presented higher percentages of *Lactobacillus* in their endometrial samples than those who suffered a miscarriage or did not become pregnant (Moreno et al., 2016). These results, together with recent findings that link low-*Lactobacillus* endometrial microbiota and infertility (Kyono et al., 2018) highlight the importance of endometrial microbiome in women's health and fertility and paves the way for new approaches based on probiotic treatments to assist women with reproductive tract dysbiosis.

In view of all these previous data, this study aimed to select a potential probiotic strain for female reproductive system health based on an ecological understanding of the problem. The considered strategy was supported by the use of probiotics to quickly lower pH and to promote the reduction of pathogenic bacteria, modifying SCFAs production and inflammation, and therefore potentially restoring the high lactobacilli-low pH healthy status. Further toxicological assays were carried out in order to assure the harmlessness of the selected strain.

## Materials and Methods

### Isolation and Identification of Strains

#### Strain Isolation

In total, 14 samples were obtained from vaginal samples. In order to obtain isolates, vaginal samples were grown on MRS medium (Oxoid) supplemented with 0.05% (w/v) of cysteine and incubated anaerobically at 37°C for 72 h. Fourteen strains were obtained from the 100 isolates recovered. Once recovered, strains were long-term stored (−20°C) in glycerol for further analysis. The isolates obtained were classified on the basis of Gram staining and cell morphology. Fourteen Gram-positive, non-sporulated rods were classified as presumptive lactobacilli and thus selected for further identification.

#### Identification and Taxonomic Characterization of Isolates

An almost complete sequence of the 16S rRNA was amplified and sequenced using an ABI PRISM BigDye Terminator Cycle Sequencing Ready Reaction Kit (Applied Biosystems Inc., Foster City, USA). DNA from pure culture was extracted using the “High Pure PCR” kit (Roche), spectrophotometrically quantified and adjusted to a final concentration of 40 ng/μl in ultra-pure water (Sigma-Aldrich, St. Louis, USA). The DNA was checked for purity, using standard methods. DNA templates were amplified by the polymerase chain reaction (PCR) on thermocycler TC-5000 (Bibby Scientific, Stone, UK), using universal primers amplifying a 1,500 bp region of the 16S rRNA gene, 616V: 5′-AGAGTTTGATYMTGGCTCAG-3′ and 630R: 5′- CAKAAAGGAGGTGATCC−3′. The amplification mixture (100 μl) comprised 2 μl (50 pmol/μl) each of 616V and 699R primers (Thermo Fisher Scientific, Waltham, USA), 0.5 μl (2 U/μl) of Taq DNA Polymerase (Finnzymes, Espoo, Finland), 10 μl of 10 × reaction buffer (Finnzymes), 10 μl of dNTP mixture containing 1 mM each of dATP, dGTP, dCTP, and dTTP (Roche Diagnostics GmbH, Penzberg, Germany), 70 μl of sterile filtered water (Milli-Q purification system, Millipore, Billerica, USA), and 5.5 μl of DNA template. The DNA template was amplified by initial denaturation at 94°C for 10 min, followed by 40 cycles of denaturation at 94°C for 1 min, annealing at 55°C for 1 min, extension at 72°C for 1 min, and a final extension at 72°C for 10 min. Controls devoid of DNA were simultaneously included in the amplification process. The integrity of PCR products was assayed by the development of single bands following electrophoresis for 1 h at 100 V in 2% (w/v) agarose gels in tris-borate EDTA buffer. Amplicons were purified using the commercial kit QIAquick PCR Purification Kit (Qiagen Inc., Valencia, USA) and subsequent sequencing reactions were performed using the Big Dye Terminator v3.1 cycle sequencing kit (Applied Biosystems), premixed format. The resulting sequences were automatically aligned and inspected by eye and compared with the on-line tool BLAST (http://blast.ncbi.nlm.nih.gov/Blast.cgi). Strains were identified on the basis of highest scores.

### *In vitro* Functional Evaluation

#### Evaluation of pH-Reducing Capacity

In order to study the capacity of lactobacilli strains to reduce pH levels, standardized cultures of the strains were grown in de Man, Rogosa and Sharpe (MRS) medium anaerobically for 17 h at 37°C. Final pH was measured with the aid of a pHmeter. Assays were performed in triplicate. After performing experiments separately, the combination of the most efficient pH-reducing strains was tested. To perform this assay, both strains were co-cultivated in MRS medium for 17 h at 37°C, and final pH was measured with the aid of a pHmeter.

#### Identification of SCFAs and Lactate Production

To obtain the organic acid profile of selected bacteria, each strain was cultured anaerobically at 37°C in 1L fermentors (Biobundle; Applikon), in the MRS. For each strain, two conditions were assayed: with and without pH control. In both cases, pH levels were monitored during the assay. Samples were collected throughout the assay.

For organic acid quantification, 0.8 mL of cell-free supernatant was mixed by vortexing with 0.2 mL of a mixture containing 5% meta-phosphoric acid, copper sulfate (1.56 mg/mL) and 50 mM 4-methyl valeric acid as an internal standard. Samples were then filtered by 0.45 μm pore size (Millipore) and diluted 1/2 and 1/10 in MilliQ water.

Organic acids were evaluated by HPLC chromatography. An aliquot of 10 μL of processed samples was injected onto a HPLC Alliance 2695 (Waters) equipped with a Rezex column [ROA Organic Acid (H+) 8% 300 × 7.8 mm, 8 um [Premium]] under conditions defined by manufacturer. Detection was achieved by an index of refraction detector (2414 Waters). The eluent was degassed H_2_SO_4_ 2.5 mM at an isocratic flow rate of 0.6 mL/min. In all cases, quantification curves were constructed adding the mixed previously defined.

### Evaluation of Antagonistic Effect of Strains on Pathogens

#### Bacterial Strains and Growth Conditions

Lactobacilli strains were grown using MRS medium supplemented with 0.05% (w/v) of cysteine, and incubated anaerobically at 37°C for 48 h. The antagonistic effect of analyzed strains was tested against *Atopobium vaginae* DSM 15829^T^, *Gardnerella vaginalis* DSM 4944, *Propionibacterium acnes* CECT 5684 and *Streptococcus agalactiae* CECT 183.

Pathogens were cultivated at 37°C anaerobically in BHI medium (Oxoid), supplemented with bovine serum (10% vol/vol; Sigma-Aldrich), and cysteine (0.5% w/vol; Sigma-Aldrich) in the case of *A. vaginae* and *G. vaginalis*. *Lactobacilli* strains were grown on MRS medium and incubated anaerobically at 37°C for 17 h.

#### Primary Cultures of Human Endometrial Epithelial Cells (hEECs)

Endometrial tissue was obtained at day 15 of the menstrual cycle from healthy donors aged 18–35 years-old. Subjects diagnosed with endometriosis and/or endometritis were excluded. The study was carried out in accordance with the recommendations of local Ethical Committee at IVI Valencia, Spain with written informed consent from all subjects. All subjects gave written informed consent in accordance with the Declaration of Helsinki. The protocol was approved by the local Ethical Committee at IVI Valencia, Spain (study code: 1404-FIVI-015-CS).

Endometrial samples were mechanically disaggregated, digested with Collagenase type A1 (Sigma) and subjected to gravity sedimentation to separate the epithelial and stromal fractions as previously described (Simón et al., 1997). The epithelial fraction was plated in 24-well plates and cultured in hEEC medium (75% DMEM, 25% MCDB-105, 10% FBS, 5 pg/mL insulin, and 0.1% fungizone and gentamicin) until they reached a confluence of 80–90%. Then, the cells were washed twice with DMEM basal media (Sigma-Aldrich) to deplete from residual antibiotic and cultured in antibiotic-free media for an additional 8 h before colonization assays.

#### Viability Assessment of hEECs

Confluent cultures of hEECs were evaluated for cell viability using flow cytometry after Propidium Iodide (PI) staining. Briefly, hEECs were collected and washed twice with phosphate buffered saline (PBS) solution and subsequently stained with 1 μg/mL of PI (Invitrogen) in staining buffer (1% BSA, 1% FBS in PBS). Cells were immediately analyzed using the 488-fluorescence laser in LSRFortessa flow cytometer (Becton Dickinson).

#### Colonization Assays

Bacterial cells from 17 h cultures were obtained by centrifugation at 4,000 rpm for 15 min. Supernatant was discarded, and pellet washed with saline solution (2 mL). Bacteria concentration was adjusted based on optical density measurements to 10^6^ cfu/mL in the case of lactobacilli and 10^4^ cfu/mL for pathogens. One milliliter of bacterial suspensions was added to each well containing confluent hECCs, and incubated at 37°C, anaerobically for 18 h. The conditions tested included individual colonization of hEECs with *L. rhamnosus* BPL005 or pathogens (*A. vaginae* DSM 15829^T^, *G. vaginalis* DSM 4944, *P. acnes* CECT 5684, and *St. agalactiae* CECT 183) and all the different combinations of the *Lactobacillus* with each pathogen. Each condition was tested in experimental duplicates and in three biological replicates.

pH vas measured with an MI-170 pH probe (Microelectrodes, Inc) before and after infection. Purity was checked microscopically, and aliquots of supernatant were recovered, centrifuged (13,000 rpm, 15 min), and both pellet and supernatant stored at −20°C until use. The supernatant fraction was used to quantify the inhibitory potential of *Lactobacillus* spp over selected populations by real time PCR, while the supernatant of the *in vitro* co-culture was used to measure pH, SCFA and inflammatory molecules (Supplementary Figure 1).

#### Quantification of Organic Acids

Short chain fatty acids acetate, propionate, butyrate together with lactate and succinate were quantified in aliquots of supernatants previously filtered through a Millipore 0.45 μm pore-size filter (Billerica, MA, USA) by HPLC chromatography. Quantification was conducted on a HPLC Acquity equipped with an Aminex HPX-87H 300 × 7.8 mm (BioRad) column under conditions defined by manufacturer. Detection was achieved by a refractive index detector. The eluent was H_2_SO_4_ 5 mM at an isocratic flow rate of 0.6 mL/min.

#### Quantification of Selected Populations by Real Time PCR

Species *A. vaginae, G. vaginalis, P. acnes*, and *St. agalactiae* were quantified by real-time quantitative PCR. DNA extraction was performed with commercial kit (Qiagen). Nucleotide sequences of primers are listed in Table 1. Oligonucleotides were purchased from Thermo Fischer (Thermo Fisher Scientific). PCR amplification and detection were performed in a StepOne Real-Time PCR System (Applied Biosystems, Foster City, Calif.), with the aid of SYBR® Green PCR Master Mix (Applied Biosystems) and Taqman Master Mix (Applied Biosystems) in the case of *P. acnes* quantification. Data were analyzed with StepOne software. In all cases, standard curves were constructed with DNA coming from 10-fold diluted cell-standardized reference cultures.

**Table 1:** Primers used in this study.

**Species**	**Primer**	**Sequence (5^**′**^-3^**′**^)**	**References**	**Concentration(nm)**	**Annealing conditions**
*G. vaginalis*	GVF	TTACTGGTGTATCACTGTAAGG	De Backer et al., 2007	200	55°C−45 s
	VR	CCGTCACAGGCTGAACAGT		200	
*A.vaginae*	AVF	CCCTATCCGCTCCTGATACC	Menard et al., 2008	200	60°C−60 s
	AVR	CAAATATCTGCGCATTTCA		200	
*S. agalactiae*	SagalactF	TTCACCAGCTGTATTAGAAGTACATGC	Van den Brand et al., 2014	500	60°C−60 s
	SagalactR	CCCTGAACATTATCTTTGATATTTCTCA		500	
*P. acnes*	PAF	GCGTGAGTGACGGTAATGGGTA	Miura et al., 2010	100	60°C−60 s
	PAR	TTCCGACGCGATCAACCA		100	
	PAP	FAM-AGCGTTGTCCGGATTTATTGGGCG-TAMRA		100	

#### Quantification of Secreted Inflammatory Molecules

Twenty-five microliters of the spent culture media from *in vitro* colonization of hEECs with bacteria were analyzed for 10 human biomarkers involved in inflammatory pathways, growth factors, chemokines, and cytokines using Luminex® Screening Assays Catalog# LxSAH (R&D Systems™) following manufacturer's instructions. Each sample was measured in duplicates. Fluorescence was read on a Luminex 200 Multiplexing Instrument.

### Safety Studies

#### Sensitivity to Antibiotics

The selected strain was tested for sensitivity to 20 antibiotics according to the European Food Safety Authority (EFSA)'s recommendations (European Food Safety Authority, 2009). The minimum inhibitory concentration (MIC) values were determined in LSM broth formulation (Klare et al., 2005, 2007) using the broth dilution antimicrobial susceptibility test established by the Clinical and Laboratory Standards Institute. The antibiotics were tested over a concentration range of 0.125 to 512 mg/liter. The assays were performed in three independent experiments.

#### Cytotoxicity in Fibroblasts

A cytotoxicity assay was performed following UNI EN ISO 10993-5 with the aid of MTT assay (Mossman, 1983) by BIOFARMA S.p.A (Italy). Assay was performed in lyophilized strain *L. rhamnosus* BPL005 with maltodextrine as a carrier. Briefly, a murine fibroblast cell line (cellule Balb/3T3, clone A31) was cultivated in Dulbecco's modified minimal essential medium (DMEM) supplemented with 10% FBS. Cell monolayers were grown in microtiter plate wells (24 h, 37°C, 5% CO_2_), washed and incubated with DMEM with decreasing concentrations of strain (from 0.5 to 0.16 mg/mL) added. Sodium lauril sulfate (0.5–0.03 mg/mL) was used as a positive control. Medium was removed, and cells incubated with 100 L/well of MTT (1 mg/ml) for 2 h at 37°C. Then MTT solution was removed and cells incubated with 200 L/well isopropanol at room temperature for 30 min. Absorbance at 570 nm and 650 nm were obtained. Cell viability (%) was calculated as [OD (570–650 nm)_sample_/OD (570–650 nm)_blank_] × 100.

#### Vaginal Irritation Tests

Vaginal irritation test was performed according to ISO 10993-10:2010. The experimental protocol followed the “Principles of laboratory animal care,” and was carried out in accordance to the European Communities Council Directive (86/609/EEC). The assay was performed by repeated applications of test sample (lyophilized strain *L. rhamnosus* BPL005 with maltodextrin as a carrier) on a group of three female albino rabbits (treated group). For this purpose, 1 mL of test sample was introduced once a day in the vagina of each animal. This procedure was repeated for five consecutive days. Another group of three female albino rabbits was treated with the same procedure (control group) using sodium chloride injection instead of test sample. Twenty-four hours after the first application and immediately prior to each administration the vaginal opening and perineum of each animal were observed for signs of discharge, erythema and oedema and results were recorded. Twenty-four hours after the last application the rabbits were sacrificed, the vaginal mucosa was dissected for histological examination.

#### Assessment of Allergic Contact Dermatitis Potential Through the Local Lymph Node Assay (LLNA)

The aim of the test is to evaluate the sensitizing potential of a cosmetic product or medical device using an *in vivo* test, the reduced Local Lymph Node Assay (rLLNA) according to the OECD 429 guideline (OECD429, 2010) and to ESAC statement on the Reduced Local Lymph Node Assay of 27th April 2007. Briefly, 8-week-old, CBA/j, female mice (Charles River, Italy) were used. Mice were divided into three groups of five animals each (5 mice per cage). Mice were housed in Standard Pathogen Free (SPF) conditions in a microbiologically controlled animal facility. Husbandry was at 20°C with 12 h continuous artificial light within each 24 h period, with 7 days for acclimatization before testing. Mice of each group were weighted at time zero (T0) and after day-6 (T6d) of the protocol.

The test products were vaginal caps. Their content was lyophilized strain *L. rhamnosus* BPL005 with maltodextrin as a carrier, which was tested following preparation of a suspension in Acetone/olive oil (AOO). The following suspension in AOO was used: 50% weight/volume. As a negative control, AOO (4/1 vol/vol) was used. As a positive control, 1-Chloro-2,4-dinitrobenzene (DNFB, Sigma-Aldrich) 0.02 % was used. Test product and controls were applied daily for 3 days (25 μl per each ear pinnae) with a micropipette. Six days following the initiation of exposure all mice receive an intravenous injection of ^3^H-labeled thymidine and 5 h later animals were sacrificed and (auricular) lymph nodes were drained and pooled for each experimental group. A single cell suspension of lymph nodes was prepared by gentle mechanical disaggregation and the cells washed and suspended in trichloroacetic acid (TCA; Sigma-Aldrich) for at least 12 h at 4°C. Precipitates were suspended in TCA and transferred to an appropriate scintillation fluid. The incorporation by draining lymph nodes of ^3^H-labeled thymidine was measured by scintillation counting and recorded as mean disintegrations per minute (dpm) for each experimental group.

For each concentration of the test material a Stimulation Index (SI) was derived relative to the concurrent vehicle control. Results for each treatment groups are expressed as the mean Stimulation Index (SI). The SI is the ratio of the mean dpm/mouse within each test product treatment group and the positive control treated group against the mean dpm/mouse for the solvent/vehicle treated control group.

### Viability With Progesterone-Based Commercial Products

To evaluate whether common used progesterone-based commercial products could have an impact on *L. rhamnosus* BPL005 viability, five commercially available products (Ultorgestan, Progeffik, Prolutex, Crinone, and Darstin) were tested against probiotic survival.

In order to test the resistance of strain BPL005 to different gynecological products, assays were performed as in the case of antibiotics resistance evaluation. In products with high turbidity, assays included a second step in which 5 μL of 24 h broth of BPL005 in the IST medium in the presence of serially diluted commercial products were dropped in agar plates to confirm the growth of the probiotic.

### Statistics

Statistical analyses were performed with IBM SPSS Statistics version 22 software, with application of univariant ANOVA and further Dunett *post-hoc* test. Significant levels were established at *p*-value ≤ 0.05.

## Results

### Isolation and Identification of Strains

Forty-four strains were obtained out of 100 isolates recovered from vagina samples. On the basis of RAPD profiles, the isolates were grouped in six different strains. Strains were identified by 16S rRNA sequencing and *Lactobacillus* strains were selected for further studies (Table 2). In addition, collection strains *L. crispatus* CECT 4840 and *L. iners* DSM 13335 were included in the study. The L. rhamnosus selected in this study on the basis of its pH-lowering activity was deposited at the Spanish Type Culture Collection under the accession number CECT 8800.

**Table 2:** Lactobacilli strains identification.

**Identification**	**Strain**
*Lactobacillus casei*	BPL013
*L. jensenii*	BPL016
	BPL017
	BPL035
	BPL044
*L. rhamnosus*	BPL005

### *In vitro* Functional Evaluation

#### Evaluation of pH-Reducing Capacity

The capacity of strains to reduce pH levels was studied. Table 3 summarizes the results obtained. In assays performed with only one strain, pH levels decreased in all cases. The highest pH reduction was obtained in the case of strain *L. rhamnosus* BPL005, being the final pH 3.82. In the case of assays with a co-culture of strains *L. casei* BPL013 and *L. rhamnosus* BPL005, the results obtained showed no differences in comparison with the results obtained with single cultures. On the basis of pH reduction results, strains *L. rhamnosus* BPL005 and *L. casei* BPL013 were selected for further studies.

**Table 3:** pH levels obtained in growth cultures of lactobacilli strains (Mean ± SD).

**Strain**	**pH**
Control (medium without culture)	6.10 ± 0.17
*L. crispatus* CECT4840	4.55 ±0.33
*L. iners* DSM13335	4.52 ± 0.51
*L. casei* BPL013	4.30 ± 0.30
*L. jensenii* BPL016	4.46 ± 0.41
*L. jensenii* BPL017	4.30 ±0.15
*L. jensenii* BPL035	4.34 ±0.37
*L. jensenii* BPL044	4.61 ± 0.34
*L. rhamnosus* BPL005	3.82 ±0.24
*L. casei* BPL13 + *L. rhamnosus* BPL005	4.25 ± 0.34

#### Identification of SCFAs and Lactate Production

**Figure 1:**
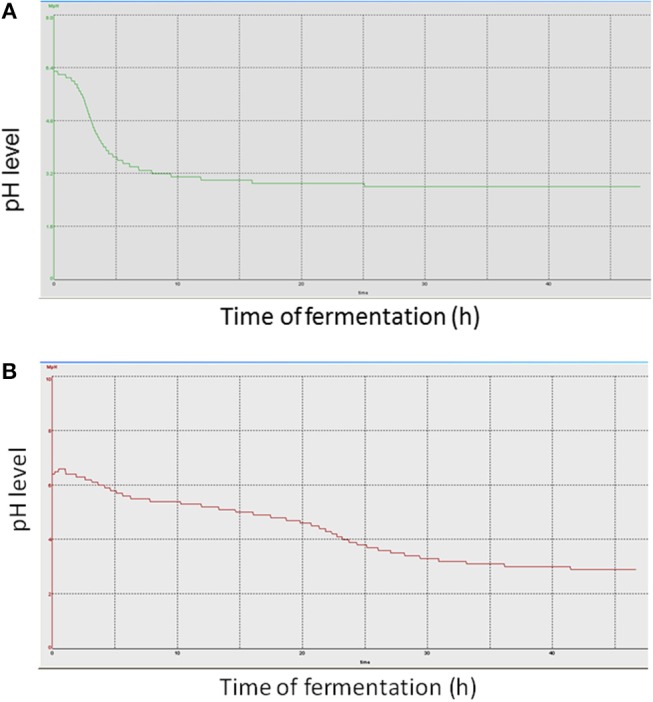
pH curve obtained in the fermentation of strains without pH control. **(A)** fermentation of BPL005; **(B)** fermentations of BPL013.

Strains BPL005 and BPL013 were analyzed on the basis of their capacity to reduce pH levels (Figure 1 shows as an example, pH monitoring of one fermentation batch per strain; all the replicates showed similar pH curves) and production of organic acids in 1 L fermentations (Tables 4, 5). In the case of strain BPL005, pH started decreasing very fast. At 5 h of fermentation, pH was 3.7 and at 10 h of fermentation pH reached its minimum (pH 3.0). In the case of BPL013 strain, pH decreased gradually, with the lowest pH (pH 3.0) at 30 h of fermentation.

**Table 4:** Levels of organic acids (g/L) obtained in BPL005 fermentations (Mean ± SD).

	**Organic acids concentration (g/L)**			
	**No pH control**			**pH control**
	**5 h**	**22 h**	**45 h**	**22 h**
Lactic acid	3.172 ± 0.066	12.168 ± 0.223^**^	15.635 ± 0.158^**^	19.731 ± 0.232^**^
Acetic acid	0.105 ± 0.050	0.123 ± 0.058	0.143 ± 0.058^**^	0.145 ± 0.049
Propionic acid	0.034 ± 0.029	0.155 ± 0.029^*^	0.210 ± 0.026^**^	0.134 ± 0.013^*^
Butyric acid	0.074 ± 0.041	0.074 ± 0.041	0.072 ± 0.039	0.082 ± 0.041
Succinic acid	0.114 ± 0.025	0.112 ± 0.042	0.157 ± 0.033	0.175 ± 0.008

*Statistics are done comparing 5 h values vs. 22 h and 45 h, respectively*.

* *p-value < 0.01*,

** *p-value < 0.001*.

**Table 5:** Levels of organic acids (g/L) obtained in BPL013 fermentations.

	**Organic acids concentration (g/L)**			
	**No pH control**			**pH control**
	**5 h**	**30 h**	**42 h**	**42 h**
Lactic acid	0.479 ± 0.090	1.387 ± 0.096^*^	10.093 ± 0.088^**^	22.814 ± 0.397^**^
Acetic acid	0.060 ± 0.051	0.065 ± 0.051	0.084 ± 0.054	0.109 ± 0.064
Propionic acid	0.058 ± 0.041	0.045 ± 0.030	0.058 ± 0.027	0.058 ± 0.023
Butyric acid	<LOQ	<LOQ	<LOQ	<LOQ
Succinic acid	0.092 ± 0.001	0.11 ± 0.002	0.104 ± 0.023	0.133 ± 0.032

*LOQ, Limit of quantification = 0.01 g/L (lactic acid, succinic acid, propionic acid, butyric acid) and 0.05 g/L (acetic acid). Statistics are done comparing 5 h values vs. 22 and 45 h, respectively*.

* *p-value < 0.01*,

** *p-value < 0.001*.

Regarding organic acids production, in the case of fermentations with controlled pH, samples were recovered at the end of the fermentation. In all cases, the highest production was obtained for lactic acid, being the maximum concentrations 22.53 g/L in the case of BPL013 and 19.90 g/L for BPL005 strain. The production of lactic acid was followed by a decrease in pH in both strains, being more remarkable in the case of strain BPL005. Regarding the production of the remaining acids, strain BPL005 was the best producer in all cases. Taking all these results into account, strain *L. rhamnosus* BPL005 was selected for further antagonistic assays.

#### Evaluation of the Effect of Strain BPL005 Reproductive Tract Pathogens Colonization

To investigate whether strain *L. rhamnosus* BPL005 might have a protective or beneficial role on endometrial infections by reproductive tract pathogens, we designed an *in vitro* model of bacterial colonization of endometrial epithelial cells. Because the co-culture of hEECs with bacteria must be performed under anaerobic conditions, we first tested the ability of hEECs to survive anaerobiosis, finding that overnight cultures of hEEC under low oxygen conditions did not significantly affect the viability of primary epithelial cells (Supplementary Figure 2).

Then, starting from endometrial biopsies of asymptomatic women, we established primary cultures of hEECs, which were cultured until they reached confluence and then colonized with pathogenic or dysbiotic bacteria previously associated to NLD microbiota (*A. vaginae, G. vaginalis, P. acnes* and *St. agalactiae*) alone or in combination with *L. rhamnosus* BPL005 for 18 h in anaerobic conditions.

Subsequently, the supernatant of the culture was used to measure pH, production of short chain fatty acids and inflammatory molecules, while the pellet containing the bacterial cells was used to assess the growth inhibition potential of *L. rhamnosus* BPL005 over dysbiotics.

#### pH Levels

Table 6 summarizes pH levels obtained in each assay. Except for streptococci infections, pH levels were not lowered by pathogen addition. When *L. rhamnosus* BPL005 was added, pH levels dropped to below pH 5. In the case of *St. agalactiae*, pathogenic strain reached levels close to those obtained with BPL005.

**Table 6:** pH levels obtained in infection endometrial assays (Mean ± SD).

**Assays**	**pH**
Control (Broth + epithelium)	6.66 ± 0.16
Control *A. vaginae*	6.63 ± 0.00
Control *G. vaginalis*	6.64 ± 0.01
Control *P. acnes*	6.22 ± 0.73
Control *St. agalactiae*	4.42 ± 0.02^a^*^^
Control BPL005	4.31 ± 0.01^a^*^^
BPL005 + *A. vaginae*	4.42 ± 0.03^b^*^^
BPL005 + *G. vaginalis*	4.43 ± 0.02^b^*^^
BPL005 + *P. acnes*	4.51 ± 0.06^b^*^^
BPL005 + *St. agalactiae*	4.41 ± 0.02

*Statistics are done*:

a *comparing control (broth + epithelium) vs. control of pathogen, are statistically different*;

b *each pathogen control vs. its grown together with strain BPL005, are statistically different*.

* *p-value < 0.001*.

#### Identification of SCFAs and Lactate Production

In the case of organic acids, differences were obtained in co-cultivation assays (Table 7). Except for *St. agalactiae* assays, the highest production was shown in the case of lactic acid, with differences in final levels (with vs. without BPL005 co-incubation) between 3.54 and 4.09 g/L. Slight production was observed for acetic acid (differences in a range of 0.04 and 0.30 g/L) and propionic acid in control BPL005 assay and co-incubated with *A. vaginae* (0.14 g/L) and *G. vaginalis* (0.14 g/L) and lower in *P. acnes* assays (−0.52 g/L). Butyric acid was not detected in any case. No significant differences were obtained for succinic acid.

**Table 7:** Quantification of organic acids after infection assays (Mean ± SD).

	**Organic acids concentration (g/L)**			
	**Acetic acid**	**Lactic acid**	**Propionic acid**	**Succinic acid**
Control (Both + Epitelium)	1.314 ± 0.062	0.399 ± 0.386	<LOQ	0.126 ± 0.002
Control *A. vaginae*	1.117 ± 0.111	0.420 ± 0.525	<LOQ	0.050 ± 0.001
Control *G. vaginalis*	1.043 ± 0.450	0.516 ± 0.410	<LOQ	0.051 ± 0.001
Control *P. acnes*	1.203 ± 0.635	0.616 ± 0.497	0.829 ± 0.869^a^*^^	0.073 ± 0.036
Control *St. agalactiae*	1.217 ± 0.246	5.172 ± 0.314^a^**^^	<LOQ	0.063 ± 0.019
Control BPL005	1.371 ± 0.122	4.769 ± 0.145^a^**^^	0.093 ± 0.002^a^*^^	0.045 ± 0.001
BPL005 + *A. vaginae*	1.325 ± 0.103	4.515 ± 0.403^b^**^^	0.139 ± 0.005^b^*^^	0.048 ± 0.001
BPL005 + *G. vaginalis*	1.338 ± 0.073	4.392 ± 0.223^b^**^^	0.142 ± 0.010^b^*^^	0.046 ± 0.003
BPL005 + *P. acnes*	1.337 ± 0.206	4.160 ± 1.189^b^**^^	0.309 ± 0.011	0.067 ± 0.024
BPL005 + *St. agalactiae*	1.264 ± 0.021	4.800 ± 0.002	<LOQ	0.048 ± 0.002

*LOQ, Limit of quantification = 0.01 g/L (lactic acid, succinic acid, propionic acid, butyric acid) and 0.05 g/L (acetic acid). Statistics are done*:

a *comparing control (broth + epithelium) vs. control of pathogen, are statistically different*;

b *each pathogen control vs. its grown together with strain BPL005, are statistically different*.

* p-value < 0.05

** *p-value < 0.001*.

#### Quantification of Selected Populations by Real Time PCR

**Figure 2:**
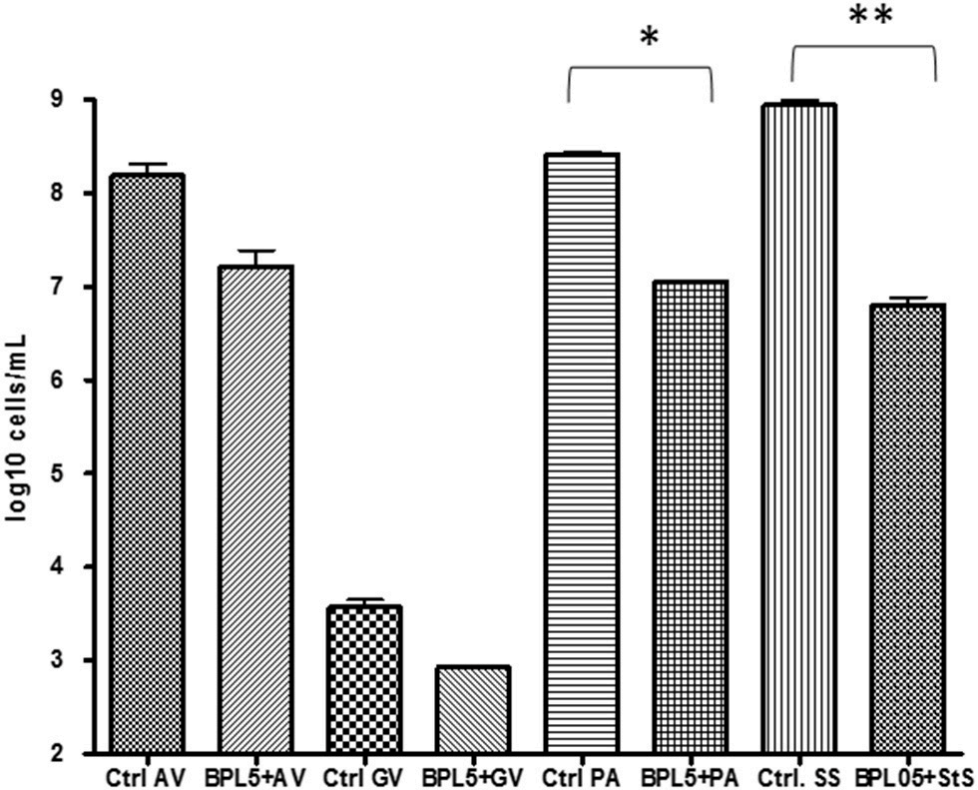
Quantification of pathogen cells in infection assays with *Lactobacillus* strains. ^*^p-value < 0.05; ^**^p-value < 0.001.

Real time PCR was used to quantify the evolution of pathogens in the infection assays (Figure 2). The co-cultivation of strain *L. rhamnosus* BPL005 with tested reference pathogens produced a significant reduction of *P. acnes* and *St. agalactiae* levels (a reduction of log_10_ 1.36 cells/mL and log_10_ 2.14 cells/mL, respectively) and a tendency but non-significant to reduce *A. vaginae* (log_10_ 0.98 cells/mL) and *G. vaginalis* (log_10_ 0.64 cells/mL).

#### Evaluation of Secreted Inflammatory Molecules

**Figure 3:**
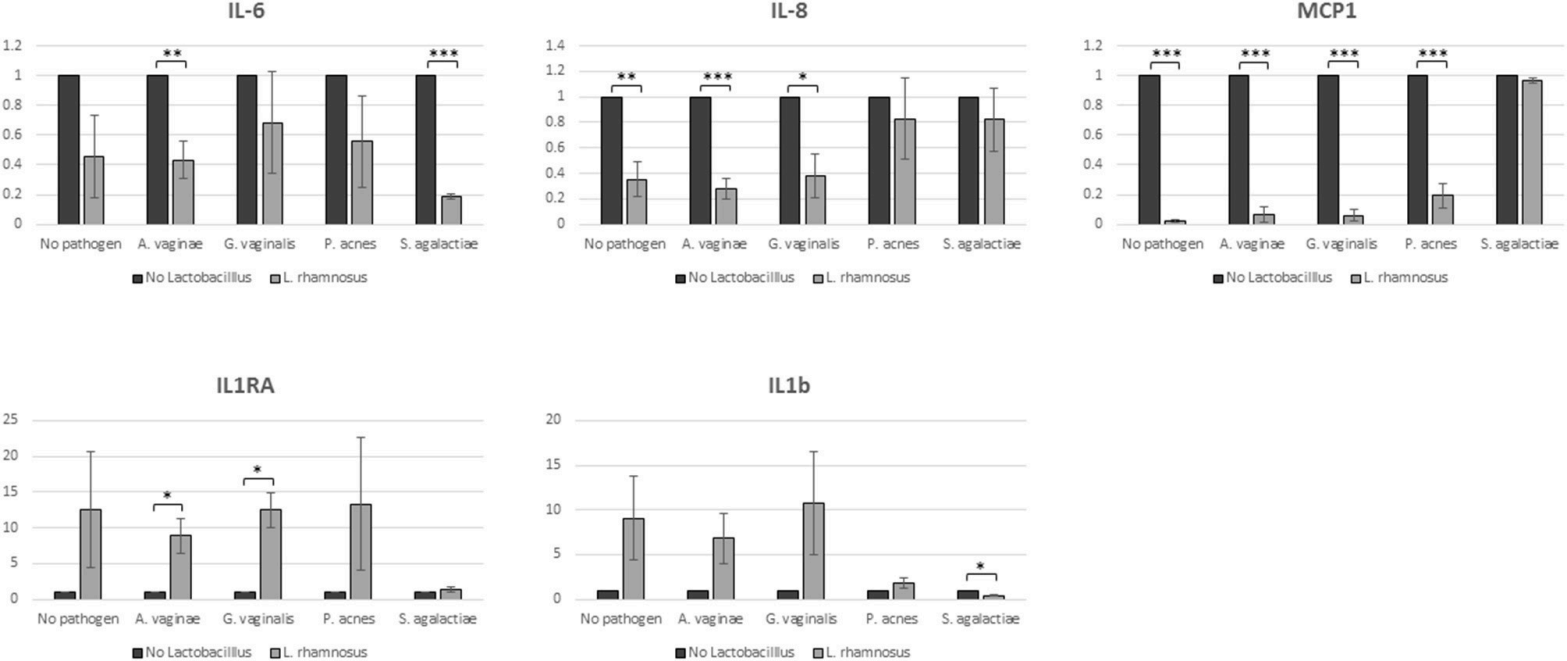
Cytokine secretion. Samples were analyzed in duplicates in 3 biological replicates (^*^p = 0.05; ^**^p = 0.01; ^***^p = 0.001).

Spent culture media obtained from co-cultures of hEECs with *L. rhamnosus* BPL005, the selected pathogens or the combinations of them, were analyzed for the secretion of cytokines, chemokines and growth factors (GM-CSF, HB-EGF, IFN gamma, IL-1 beta, IL-1 RI, IL-1 RA, IL-6, IL-8, MCP-1, and RANTES). No detectable levels of GM-CSF, HB-EGF, IFN gamma, and IL-1 RI were recorded in our assay and these molecules were excluded from the analysis. The average concentrations of the rest of molecules secreted to spent media by hEECs under control conditions (before colonization with bacterial cells) were: IL-6, 868.25 pg/mL; IL-8, 29.81 μg/mL; MCP-1, 1.99 μg/mL; IL-1 beta, 9.39 pg/mL; RANTES, 10.67 pg/mL, and IL-1 RA, 643 pg/mL. The amount of RANTES secreted by hEECs did not show changes upon colonization with bacteria, either *L. rhamnosus* BPL005, dysbiotic, pathogenic bacteria, or combinations between them. The colonization of *L. rhamnosus* BPL005 in the culture produced a decrease in IL-6, IL-8, and MCP-1, that was much more evident in the presence of pathogens, and presented significant values in the case of co-cultivation with *A. vaginae* (IL-6: 2-fold decrease, *p* < 0.01; IL-8: 3.5-fold decrease, *p* < 0.001; MCP-1: 16.5-fold decrease, *p* < 0.001), *G. vaginalis* (IL-8: 2.6-fold decrease, *p* < 0.001; MCP-1: 16.5-fold decrease, *p* < 0.001), *P. acnes* (MCP-1: 5-fold decrease, *p* < 0.001), and *St. agalactiae* (IL-6: 5-fold decrease, *p* < 0.001). On the other hand, addition of *L. rhamnosus* BPL005 to the culture produced a generalized increase in IL-1RA and IL-1 beta, showing statistically significant increase in the presence of *A. vaginae* and *G. vaginalis* (IL-1 RA: 8.9 and 12.5-fold change increase respectively, *p* < 0.05) and *St. agalactiae* (IL-1 beta: 2.5-fold change, *p* < 0.05) (Figure 3).

### Safety Assessment

#### Antibiotic Resistance

Antibiotic resistance assays were performed in BPL005 strain and MIC values are shown in Table 8. In the case of chloramphenicol and erythromicin, MIC values of BPL005 were higher than EFSA breakpoint, but similar to and lower than MIC values obtained, respectively, for commercial strain LGG.

**Table 8:** Antibiotic resistance of strain BPL005 expressed as Minimum Inhibitory Concentration.

	**MIC (μg/ml)**	
	**BPL005**	**Breakpoint**^**a**^
Amoxicillin	4	n.r.
Ampicilin	4	4
Azithromycin	>256	n.r.
Carbencillin	32	n.r.
Clarithromycin	8	n.r.
Clindamycin	1	1
Gentamicin	4	16
Kanamycin	64	64
Metronidazole	64	n.r.
Nalidixic acid	>256	n.r.
Oxytetracycline	16	n.r.
Penicillin	8	n.r.
Polymyxin B	>256	n.r.
Rifampicin	2	n.r.
Streptomycin	16	32
Sulfonamide	>256	n.r.
Tetracycline	8	8
Trimethoprim	128	n.r.
Vancomycin	>256	n.r.

*n.r., No obligatory in the case of L. rhamnosus strains for EFSA*;

a *EFSA Breakpoint (European Food Safety Authority, 2012)*.

#### *In vitro* and *in vivo* Safety Assays on the Final Product

Evaluation of safety was performed on a final product, defined as a vaginal capsule. Cytotoxicity assays in fibroblasts showed no cytotoxic effect for any of the concentrations tested (Table 9). In the case of vaginal irritation tests, none of the animals suffered irritation at macroscopic and microscopic level, being considered as minimally irritant. Finally, in the assessment of allergic contact dermatitis potential through the Local Lymph Node Assay (LLNA, Table 10), no signs of general toxicity were observed. Neither weight loss nor signs of local irritation were observed in any animals, including the DNFB-treated mice. The latter observation was likely due to the relatively low concentration (0.02%) of DNFB used. DNFB showed an evident sensitizing effect, as expected.

**Table 9:** Cytotoxicity assays.

SLS (POSITIVE CONTROL)^*^						
Dosage (mg/mL)	0.5	0.25	0.13	0.06	0.03	
% cell vitality	1.9	1.1	12.6	1,112.9	106.7	
Standard deviation	0.3	0.3	5.6	0.3	2.6	
VAGINAL CAPSULES WITH BPL005 ADDED^**^						
Dosage (mg/mL)	5	0.25	1.25	0.63	0.31	0.16
% cell vitality	88	78.9	76.4	82.9	78.9	76.2
Standard deviation	1.1	0.5	0.2	2.5	1.5	1.3

* *IC50 = 0.10 mg/mL. Cell mortality was >30% in the first 3 dilutions tested*.

** *IC50 ≥ 5 mg/mL. Cell mortality was <30% in all the dilutions tested*.

**Table 10:** Assessment of allergic contact dermatitis potential through the Local Lymph Node Assay.

**Mouse number**	**Disintegrations per minute (dpm)**		
	Negative control^*^	Positive control^**^	Treatment^***^
1	99	357	75
2	105	425	105
3	56	325	100
4	98	333	95
5	112	298	99
Mean ± SD	94 ± 22	348 ± 48	95 ± 12

* *Acetone/olive oil (4:1 vol/vol) S.I. = 1*;

** *DNFB 0.02%, S.I. = 3.70*;

*** *Vaginal caps with probiotics, S.I. = 1.01*.

### Viability With Progesterone-Based Commercial Products

To evaluate whether commonly used progesterone-based commercial products could have an impact on *L. rhamnosus* BPL005 viability, five commercially available products (Ultorgestan, Progeffik, Prolutex, Crinone, and Darstin) were tested against probiotic survival. In all cases, strain BPL005 was resistant to the highest concentration tested and no inhibitions were observed, even at the highest concentrations in gel and cream-based products (data not shown).

## Discussion

The impact of the microbiota on human health and disease has gained attention in the last decade, with research revealing the importance of the microbial ecosystem in different body sites and the role of host-microbial interactions in physiological functions.

The female reproductive tract is reported to be mainly colonized by *Lactobacillus* species in healthy subjects of reproductive age, while the significant presence of dysbiotic or pathogenic bacteria is associated with disease, including cancer, reproductive dysfunction and obstetric complications (reviewed by Moreno and Franasiak, 2017). Accordingly, the restoration of optimal bacterial community in women with altered genital microbiota is an advisable approach to improving clinical management of several conditions. Traditionally, problems arising from dysbiosis have been treated with antimicrobial drugs, which tackle the problem short-term but, in most cases, aggravate the underlying dysbiosis mid- and long-term and may promote resistance. Our current understanding of the role of balanced microbiota in health and disease has given rise to other approaches aiming to correct the factors influencing this balance and its subsequent consolidation. Among the different strategies available to modulate the microbiota, the use of live biotherapeutic products is very attractive given that it attacks the problem from the base by trying to correct the source of the imbalance from an ecological approach, not only affecting the specific problem, but also lowering the possibility of recidivism. Moreover, biotherapeutic products exhibit low toxicity and side effects. Following this rationale, although the use of probiotics has been applied traditionally in digestive health, now these microorganisms are considered the most promising area of work within biotherapeutics. In fact, the administration of vaginal probiotics alone or complementary to antibiotic therapy improves vaginal health and avoids recurrence of BV (reviewed by Tachedjian et al., 2017; Anahtar et al., 2018).

The colonization rate of the cervicovaginal communities after one cycle of probiotic treatment ranges between 10 and 59%, depending on the species of *Lactobacillus*, the via of administration (oral or vaginal), and other individual-dependent variables (Antonio et al., 2009; Hemmerling et al., 2010; Bohbot and Cardot, 2012). However, despite studies showing the ability of *Lactobacillus* probiotics to colonize the vagina, increasing counts of this bacteria in the reproductive tract, their beneficial effects on reproductive or obstetrical outcomes are still questioned (Gilboa et al., 2005; Gille et al., 2016). This lack of effectivity could be the consequence of low colonization rates of probiotic strains isolated from niches other than the reproductive tract and also the use of preclinical screening studies based only on antagonist analysis against pathogens. In our study, we worked with vaginal isolates taken from the female reproductive tract of healthy women, on the rationale that isolating strains from this environment may increase their successful adaptation and colonization of this niche when administered clinically. The workflow was designed with strain selection first based on their ability to sharply reduce pH and to produce SCFAs, followed by an *in vitro* model of pathogen colonization of endometrial epithelial cells with selected strains. These assays analyzed the ability of the probiotic strains to act against pathogen colonization and modify SCFA production and inflammation-related molecules.

As a first step, six vaginal isolates and two reference strains were screened based on their capacity to lower pH. Among all the strains tested, two *L. rhamnosus* (BPL005 and BPL013) were selected. These strains showed final pH levels below 4.5 and no differences were obtained when both were combined. Subsequently, the ability of these two strains to reduce pH was compared in depth using growth assays with monitored pH, in which strain BPL005 reached the minimum pH at 10 h of fermentation. Although past reported probiotics selection has been based on the production of direct antimicrobial activity molecules (i.e., H_2_O_2_, bacteriocins) and adhesion, in our case the capacity of lactobacilli to acidify the environment was our screening variable. The beneficial role of lactobacilli on acidification has been reported, related with healthy Nugent scores and a balanced microbiome (Ravel et al., 2011). This strong acidification potential is mainly due to lactic acid production, with widely reported positive effects including the control of pathogen populations, immune modulation and colonization (Borges et al., 2014). In our case, even though both strains BPL005 and BPL013 produced mainly lactic acid, with levels close to 20 g/L, strain BPL005 produced the highest levels faster, which coincides with its high capacity to reduce pH quickly, and thus it was selected for further study.

Subsequently, we analyzed the activity of the BPL005 strain on the colonization of pathogenic or dysbiotic bacteria previously associated to NLD microbiota (*A. vaginae, G. vaginalis, P. acnes*, and *St. agalactiae*) in the endometrial epithelial model. The model developed here starts with individual endometrial biopsies of asymptomatic women and mimics normal endometrial epithelium with the aim of bringing the model as close as possible to the environment in which the probiotic should perform its function. Using this model, the capacity of strain BPL005 to lower pH was confirmed, being pH levels below pH 5.0 when co-cultures of BPL005 plus pathogens were assayed. In the case of *St. agalactiae*, as this species is also a lactic acid producer, pH dropped in both conditions with and without probiotic co-culture and no differences were obtained. Two major findings resulted from analyzing organic acid production. Propionic acid was produced when *P. acnes* strain was incubated with endometrial cells, as expected on the basis of its metabolic activity and, when the pathogenic species *P. acnes* was co-incubated with BPL005, levels were reduced. A drop in propionic acid levels can lead to a drift toward a healthy organic acid profile, as this compound has been linked to symptomatic BV profiles (Yeoman et al., 2013). Moreover, there was high production of lactic acid in co-cultivation assays. Beyond the effect on the drop in pH, this organic acid has demonstrated a strong bactericidal effect against pathogens colonizing the reproductive system (O'Hanlon et al., 2010). In fact, recent studies indicate effects are specifically related with lactic acid presence, thus reducing the plausibility of the H_2_O_2_ effect on pathogens physiological conditions (Ocaña et al., 1999a; O'Hanlon et al., 2011).

In order to investigate whether these changes in the profile of organic acids and pH values result in differences in pathogen colonization, we analyzed the influence of *L. rhamnosus* BPL005 on pathogen colonization by species-specific real-time PCR. Results showed an effect of *L. rhamnosus* BPL005 against *P. acnes* and *St. agalactiae* levels and lower but non-significant levels of *A. vaginae* and *G. vaginalis*. As assays have been conducted with individual pathogenic strains, the potential interactions among them, or how the *L. rhamnosus* BPL005 strain could interact in a disbiotic pathogenic niche, including both bacteria and yeast inhabitants, cannot be extrapolated with our results. As an interest-worthy fact at this point, it is worth mentioning that a clinical study has been carried out with the strain, showing positive effect in lowering bacterial pathogens (unpublished data). In addition to organic acid production and low pH, there are other theoretical pathogen inhibition pathways that may be exerting an effect on the pathogens tested. Regarding the production of bacteriocins and H_2_O_2_, in our study we did not analyze whether the effect observed against the pathogens came only from the production of organic acids and lowering pH or if these molecules exerted an action against the pathogens tested. In the case of H_2_O_2_, the production was again not checked. Although hydrogen peroxide has traditionally been considered a molecule responsible at least in part for the action against pathogens, its *in vivo* activity is questioned (Tachedjian et al., 2018). Regarding the potential effect of bacteriocins, the production of this type of substances has been observed in strains of the species *L. rhamnosus* with activity against vaginal pathogens (Ruíz et al., 2012), so that, although not evaluated in this work, its action would not be neglected. Although the effect of the strain on pathogens maybe partly attributable to antimicrobial and anti-adhesive compound production, as already observed for other *Lactobacillus* strains (Coudeyras et al., 2008; Petrova et al., 2016; Bertuccini et al., 2017), we must also consider the effect that pH has on the state of pathogens and therefore on their ability to grow and adhere to endometrial cells. This blocking of pathogen adhesion and lowering of pH may provide an advantage to the communities adapted to this environment and therefore return to and establish a balanced and healthy system. In the upper genital tract, it is still uncertain how the microbiota and pH impact on the health status, and on how vaginal microbiota affects pH, microbial population and subsequently health, but it is putatively related with genomic stability, epithelial barrier, microbial-secreted metabolites and inflammation (Baker et al., 2018). Recent findings have also found a structured polymicrobial *G. vaginalis* biofilm in the uterus of women with BV (Swidsinski et al., 2013), pointing to a potential incidence of vaginal pathogens on upper-genital microbial condition. In our case, the exposition of pathogens to *L. rhamnosus* BPL005 blocked their colonization of hEECs conferring a potentially positive effect on biofilm inhibition. As exposed above, these changes in microbiota composition should have an effect on inflammatory markers. Through the production of lactic acid *Lactobacillus* ssp. can promote an immunomodulatory function on the reproductive tract. In this regard, *Lactobacillus* has been shown to decrease the pro-inflammatory molecules like IL-6, IL-8, MIP-3α, RANTES, and TNFα, while increasing the secretion of the anti-inflammatory cytokine IL-1RA (Hearps et al., 2017; Tachedjian et al., 2017). Soluble factors such as cytokines and chemokines are known to play a pivotal role in reproduction. For example, pro-inflammatory cytokines as IL-6, IL-8, LIF, and TNF-α increase from the proliferative to the luteal phase to prepare the endometrium for embryo implantation (Benner et al., 2018), while CSF-1, GM-CSF, HB-EGF, IGF-1, IGF-2, and LIF improve fertility by helping blastocyst development (Robertson and Moldenhauer, 2014). In our study, we found that colonization of hEECs with different dysbiotic/pathogenic bacteria modulated the secretion of different cytokines and chemokines compared to colonization with BPL005, showing that an NLD microbiota might trigger an inflammatory response in the endometrium characterized by increased proinflammatory cytokines IL-6, IL-8, and MCP-1. Also, for some of these molecules (IL-6, IL-8, MCP-1, IL-1RA, and IL-1b), addition of BPL005 to hEECs colonized with pathogens, significantly restored equivalent levels to the *Lactobacillus*-only colonization, suggesting a potential role of this BPL005 probiotic to decrease the levels of pro-inflammatory cytokines produced by an NLD microbiota and maintain the inflammatory homeostasis of endometrial epithelial cells by increasing the levels of the anti-inflammatory molecule IL-1RA. This is clinically relevant because alterations in the cytokine and/or chemokine levels have been related to infertility. IL-6 is one of the essential cytokines in embryo implantation, and women with unexplained infertility and/or endometriosis present increased levels of secreted IL-6 in the endometrium and other reproductive sites, indicative of an unfunctional endometrium, as this increased IL-6 is also observed in women carrying intrauterine devices (Ammala et al., 1995; Tseng et al., 1996). Also, elevated IL-8 and MCP-1 are found in peritoneal fluid of patients suffering from endometriosis compared to controls and their levels correlate with the severity of the disease (Arici et al., 1996; Zeyneloglu et al., 1998). IL-8 could promote proliferation and adhesion of endometrial cells outside the uterus, a hallmark of this inflammatory disease (Arici, 2002). Interestingly, elevated levels of pro-inflammatory cytokines have been described in gynecological diseases of infectious origin, as is the case of high MCP-1 in abdominal-pelvic adhesions (Zeyneloglu et al., 1998), or increased IL-1b, IL-6, and TNFα in menstruation from infertile patients with chronic endometritis (Tortorella et al., 2014), supporting the implication of bacterial pathogens causing CE in the growth of these pro-inflammatory molecules in the endometrium. It has been also described that endometrial secretion of IL-6 is stimulated by increasing concentrations of IL-1b in stromal but not in epithelial cells (von Wolff et al., 2002). However, for some other molecules, the interrogated cytokines were undetectable in our system (GM-CSF, HB-EGF, IFN gamma, and IL-1 RI) or did not show significant differences between the colonization of primary hEECs with healthy or dysbiotic bacteria (RANTES). This could be due to the individual variability among the tissue samples used to establish hEECs.

Finally, and in order to ensure the safety of the *L. rhamnosus* BPL005 strain, a detailed toxicological study was carried out. Safety assays included in this work have concluded the absence of cytotoxic effects and irritation at macroscopic and microscopic levels, being considered as minimally irritant. In the case of cytotoxicity in fibroblasts, it is necessary to point out that MTT is not specific for eukaryotic cells, and these results should be validated with other metrics or study. Finally, no signs of general toxicity were observed in the assessment of allergic contact dermatitis potential through the Local Lymph Node Assay.

Based on all these results, the proposed intervention strategy using the *L. rhamnosus* BPL005 strain and supported by a rapid drop in vaginal pH can be considered as an effective strategy to resolving bacterial imbalance and to subsequently re-establish healthy profiles with consequent positive effects on the upper-genital tract. This strategy can provide greater benefit than conventional probiotics, which usually achieve a partial inhibition of the pathogens present and, as the problem of environment is not resolved (high pH, probiotic strain not adapted to genital tract), microbiota continues unstable and infections and related discomforts recur. Further clinical studies should be addressed to demonstrate in a final commercial matrix (probiotic ovules) its capacity of colonizing and moreover its effect on microbiome, pH and health parameters in the genital tract.

## Ethics Statement

The study was carried out in accordance with the recommendations of local Ethical Committee at IVI Valencia, Spain with written informed consent from all subjects. All subjects gave written informed consent in accordance with the Declaration of Helsinki. The protocol was approved by the local Ethical Committee at IVI Valencia, Spain (study code: 1404-FIVI-015-CS).

## Author Contributions

EC, IM, CS, and DR designed the research. MS, IG-G, AS, and MG-M performed the research. EC, IM, FV, and SG analyzed the data. CS-D, CS, and DR evaluated the data. EC and IM wrote the manuscript. EC, IM, CS-D, CS, and DR had primary responsibility for the final content. All authors read and approved the final manuscript.

### Conflict of Interest Statement

EC, SG, and DR are employed by Biopolis; IM, MG-M, and CS are employed by Igenomix; CS-D is employed by Ferring Pharmaceuticals. The remaining authors declare that the research was conducted in the absence of any commercial or financial relationships that could be construed as a potential conflict of interest.
