# Supplementary material for: Selection of New Probiotics for Endometrial Health
Source: Front Cell Infect Microbiol. 2019 Apr 17;9:114. doi: 10.3389/fcimb.2019.00114 (PMC6481279; doi:10.3389/fcimb.2019.00114)
Supplement: Supplementary file 1 [file Presentation_1.PPT]

## Slide 1
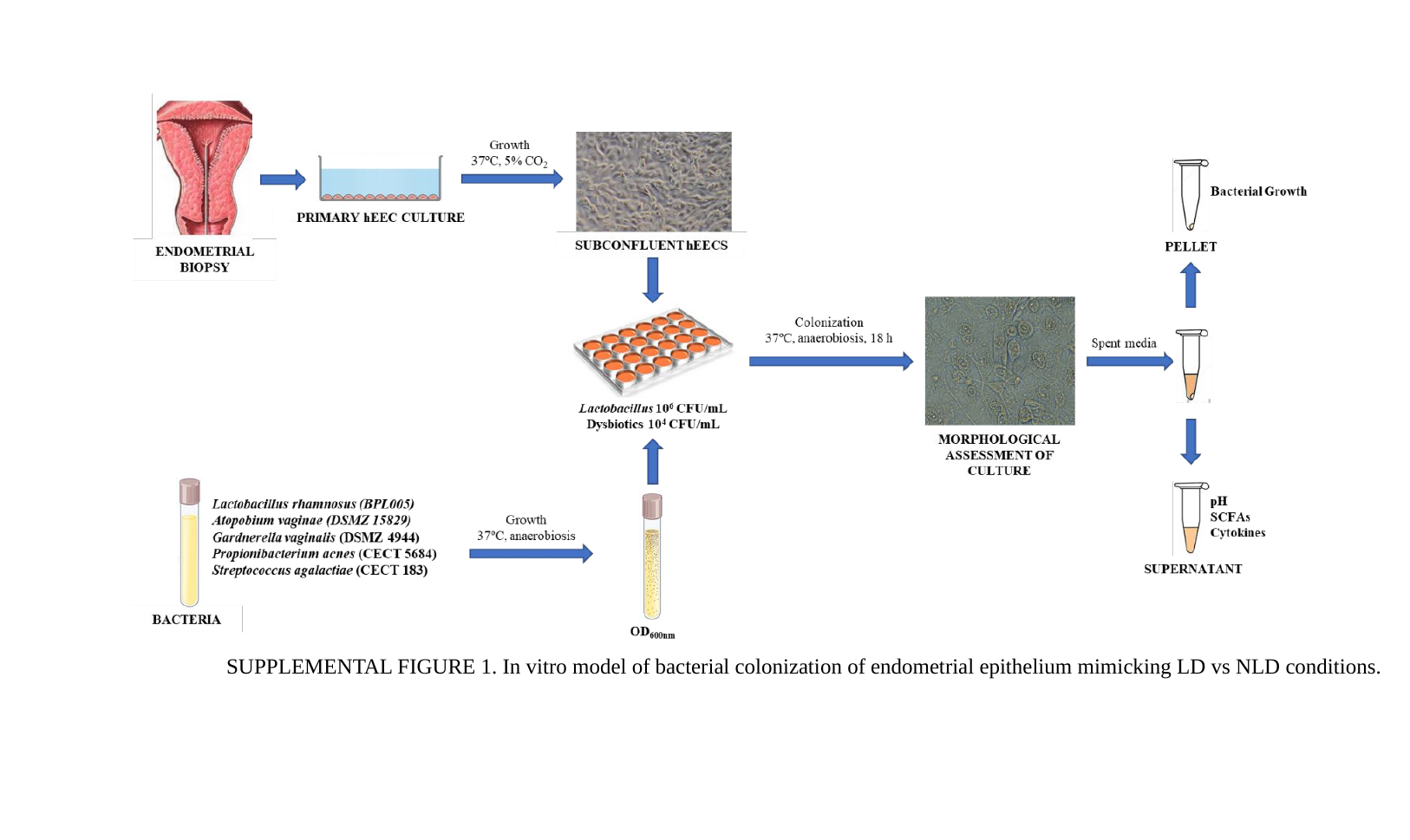

SUPPLEMENTAL FIGURE 1. In vitro model of bacterial colonization of endometrial epithelium mimicking LD vs NLD conditions.
