# Supplementary figures and images for: Selection of New Probiotics for Endometrial Health
Source: Front Cell Infect Microbiol. 2019 Apr 17;9:114. doi: 10.3389/fcimb.2019.00114 (PMC6481279; doi:10.3389/fcimb.2019.00114)

## Slide 1
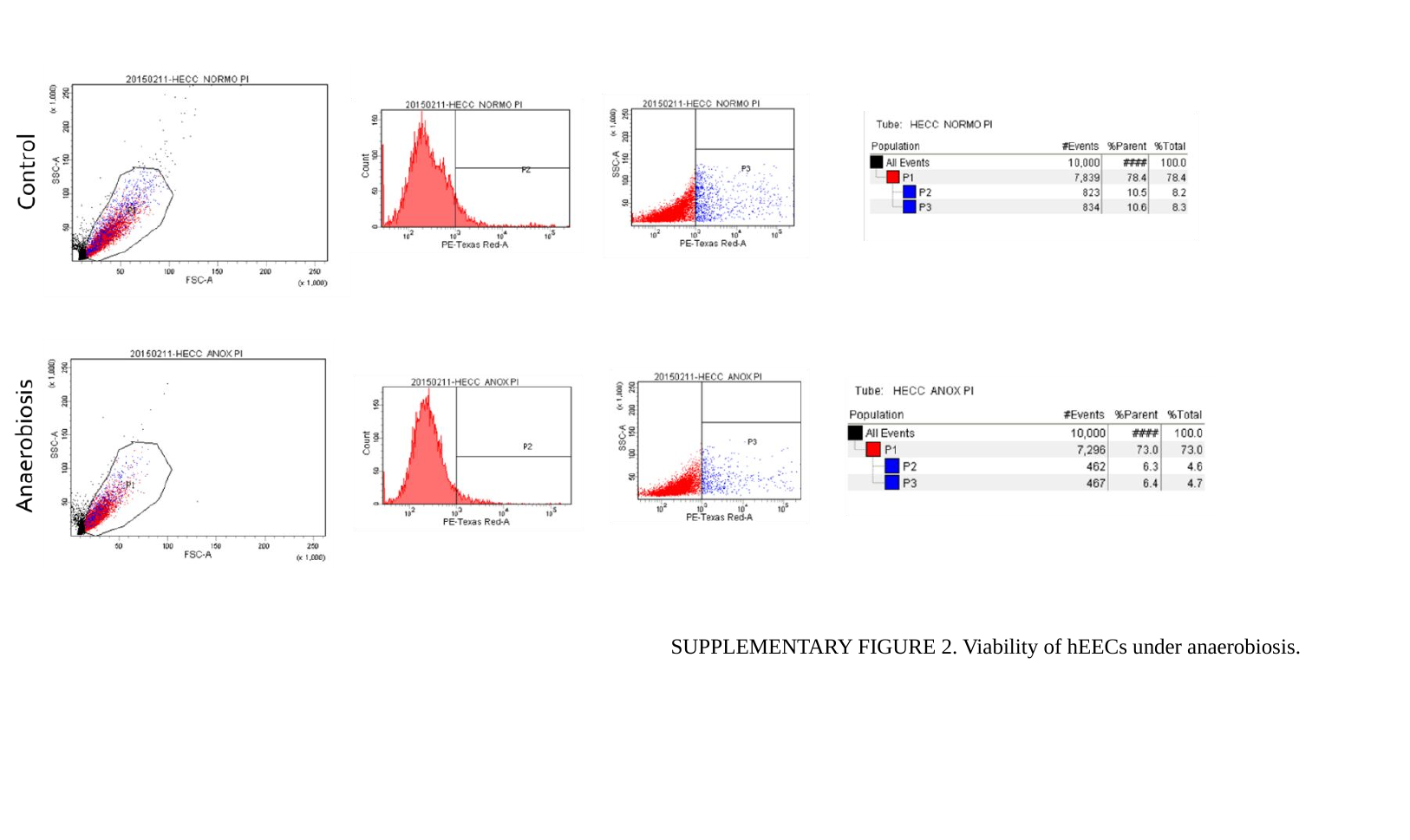

SUPPLEMENTARY FIGURE 2. Viability of hEECs under anaerobiosis.

Supplement: Supplementary file 2 [file Presentation_2.PPT]
